# Teaching and evaluating multitasking ability in emergency medicine residents - what is the best practice?

**DOI:** 10.1186/s12245-014-0041-4

**Published:** 2014-09-26

**Authors:** Kenneth WJ Heng

**Affiliations:** 1 Emergency Department, Tan Tock Seng Hospital, 11 Jalan Tan Tock Seng 308433, Singapore

**Keywords:** Multitasking, Medical education, Evaluation

## Abstract

Multitasking is an essential skill to develop during Emergency Medicine (EM) residency. Residents who struggle to cope in a multitasking environment risk fatigue, stress, and burnout. Improper management of interruption has been causally linked with medical errors. Formal teaching and evaluation of multitasking is often lacking in EM residency programs. This article reviewed the literature on multitasking in EM to identify best practices for teaching and evaluating multitasking amongst EM residents. With the advancement in understanding of what multitasking is, deliberate attempts should be made to teach residents pitfalls and coping strategies. This can be taught through a formal curriculum, role modeling by faculty, and simulation training. The best way to evaluate multitasking ability in residents is by direct observation. The EM Milestone Project provides a framework by which multitasking can be evaluated. EM residents should be deployed in work environments commiserate with their multitasking ability and their progress should be graduated after identified deficiencies are remediated.

## 1 Review

### 1.1 Introduction

Multitasking is a core skill in emergency medicine (EM). Emergency department (ED) work is by nature susceptible to interruptions due to time-critical tasks, patients arriving unpredictably in surges with undifferentiated problems with varying acuity. In addition, universal problems like access block and ED overcrowding add to the multitasking duties of the EM physician.

The ability to multitask does not come naturally to everyone, and often there is no formal curriculum to teach residents how to cope in the multitasking ED environment. Assuming that all residents will automatically assimilate and passively develop this skill during residency is flawed. There are multiple consequences of not recognizing and helping residents who struggle to cope within the multitasking environment - physician job stress, burnout [1], fatigue, and sleep deprivation [2]-[4]. Archana et al. [5] described patient safety lapses due to gaps in information flow as a result of deficiencies in multitasking, and the Institute of Medicine describes interruptions as contributing factors to medical errors [6]. Furthermore, there is often no formal evaluation of a resident’s ability to multitask with the faculty often relying on subjective intuition and ‘gut feel’. The aim of this article is to review the literature on multitasking in EM and to identify best practices for teaching and evaluating this skill amongst EM residents.

### 1.2 Methods

The reviewed articles’ inclusion criteria were as follows: (1) the setting was healthcare in general and the emergency department in particular; (2) the main focus was on teaching or evaluating multitasking, interruptions, or task switching; and (3) the article was available in the English language. Articles were excluded if they only contained conceptual or theoretical discussions of multitasking.

The online databases PubMed, MEDLINE, and CINAHL were searched using the following search phrases: {multitask* OR interruption OR task switch*} AND {health care* OR emergency*}. These articles used the terms interruption, break-in-task, task switching, and multitasking interchangeably. ‘Interruption’ was subsequently disregarded because it provided too many irrelevant articles. In addition, a search of the websites of the Council of Emergency Medicine Residency Directors and the Accreditation Council for Graduate Medical Education, and American Board of Emergency Medicine was made for relevant publications. The themes from the articles reviewed are categorized below.

### 1.3 Results

This search yielded a total of 29 articles. A search of their references yielded five additional articles meeting the inclusion criteria.

#### 1.3.1 Definition of multitasking in the context of emergency medicine

The definition of multitasking in EM has evolved (Table 1). In the 2011 update on the Clinical Practice of EM [7], multitasking was defined as the ability to prioritize and implement the evaluation and management of multiple patients in the emergency department, including handling interruptions and task switching in order to provide optimal care. This is the first time that the Clinical Practice of EM has explicitly broken down multitasking into interruptions and task switching and this differentiation has been maintained in the 2013 update.

**Table 1:** Definition of multitasking from the ‘Model of the clinical practice of emergency medicine’

	**Definition**
2009	Multitasking and team management
	Prioritize multiple patients in the ED to provide optimal patient care; interact, coordinate, educate, and supervise all members of the patient management team; utilize appropriate hospital resources; and have familiarity with disaster management.
2011, 2013	Multitasking (team management is separately described)
	Multiple patient care:
	Prioritize and implement the evaluation and management of multiple patients in the emergency department, including handling interruptions and task switching, in order to provide optimal patient care.

#### 1.3.2 Extent of multitasking in EM practice

Three time-motion studies set in the ED report that EM physicians receive between 9.7 and 11.1 interruptions per hour [8]-[10]. In another study, Archana et al. found an average of one interruption every 9 and 14 min for EM physicians and residents, respectively [5]. Research by Chisholm et al. [11] described that EM physicians are ‘interrupt-driven’ and that these frequent interruptions result in breaks-in-task.

The frequency of interruptions and breaks-in-task encountered were correlated with the average number of patients simultaneously managed. Each break-in-task raises the potential for errors occurring. In another time-motion study, consultants performed 101 discrete tasks per hour, with 77 min of overlapping activity, broken down into 42% of each hour spent on communication, 35% on direct clinical care, and 24% on computer use; and 9% on non-clinical tasks [12].

When comparing interruptions between EM physicians and primary care physicians, Chisholm et al. [9] found that the former were more frequently interrupted. The reason cited for this was the work environment, where EM physicians were more likely to interact with more healthcare workers, taking care of unscheduled patients, thus increasing the likelihood of interruptions as opposed to primary care physicians who are primarily office-based and care for scheduled patients.

#### 1.3.3 Teaching residents how to cope in a multitasking environment

A method recommended for teaching multitasking is via real-time role modeling by faculty and senior residents [13]. Another method for teaching residents to multitask is through simulation training. High-fidelity simulators in mock-ups resembling the ED involving multiple patients and multidisciplinary team have been shown to improve clinical abilities, specific cognitive strategies, and teamwork [14]. By controlling scenario complexity, resource limitation, and scripted interruptions, simulation training can challenge the resident’s ability to adapt and improvise in a safe environment. Proper post-scenario team debrief is essential to encourage individual reflective learning.

#### 1.3.4 Evaluation of EM residents’ multitasking ability

The multitasking assessment tool (MTAT) was utilized at the time of resident selection, and this multitasking ability score was compared to subsequent work efficiency [15]. It was found that the variance in work efficiency was correlated to a large extent by years of training and to a smaller extent by the MTAT score.

For evaluation by direct observation, the Council of EM Residency Directors Workgroup on teaching and evaluating difficult-to-teach competencies listed sign-out rounds and multidisciplinary team simulation as high-yield activities to evaluate multitasking ability [13]. Several criteria to evaluate multitasking ability were published (Table 2).

**Table 2:** **Multitasking - focused residency evaluation criteria (Wang**[13]**)**

	**Evaluation criteria**
Charting	Is the resident’s charting accurate, complete, and timely?
	What is the resident’s number of patients seen per hour and length of stay data relative to peer group?
	Does the resident practice patient follow-up and reassessment during ED evaluation?
Prioritization	Does the resident appropriately prioritize tasks?

Of the two most commonly used direct observation tools currently available, such as the mini clinical examination (mini-CEX) [16] and the standardized direct observation tool for EM (SDOT) [17], the SDOT comes the closest to evaluating multitasking ability. In SDOT, two anchors describe the following desirable traits: ‘Prioritizes patients appropriately by acuity and waiting time’ and ‘Controls distractions and other priorities while maintaining focus on patient’s care’.

A joint project entitled ‘The Emergency Medicine Milestone Project’ by the Accreditation Council for Graduate Medical Education and the American Board of Emergency Medicine [18] described competency-based developmental outcomes that can be tracked as the resident progresses from the beginning of their education, through graduation, to unsupervised practice. Table 3 lists descriptors for each of the five milestones in multitasking ability. This is a useful framework with which to evaluate and benchmark a resident’s multitasking ability.

**Table 3:** Milestones in multitasking (task switching)

**Milestones in multitasking**	
Level 1	Manages a single patient amidst distractions
Level 2	Task switches between different patients
Level 3	Employs task switching in an efficient and timely manner in order to manage multiple patients
Level 4	Employs task switching in an efficient and timely manner in order to manage the ED
Level 5	Employs task switching in an efficient and timely manner in order to manage the ED under high-volume or surge situations

Finally, high-stake summative EM examinations currently evaluate multitasking ability: the multiple patient encounters in the ABEM oral boards and ‘management in-tray exercise’ in the Fellowship of the College of EM (United Kingdom) exit examination. Clearly, the highest EM authorities in both countries regard multitasking as an essential skill that senior trainees about to embark on independent practice must possess.

### 1.4 Discussion

The study of human factors is the discipline which addresses human behavior, abilities, limitations, and relationship with the work environment. The human factors framework describes an intricate interplay between human factors (other healthcare workers, shift work, handovers, staffing, authority gradients, polices and protocols, training and supervision of residents) with the physical environment that influences an individual’s performance. This interplay of factors act together to produce effects and behaviors such as distractions, interruptions, fatigue, and stress [19].

Psychologists believe that the human brain is incapable of multitasking, that we cannot focus on more than one thing at a time, especially when tasks require the same cognitive domain. Instead, when we ‘multitask’ , we are actually switching focus rapidly between tasks rather than completing them simultaneously. Therefore, multitasking appears to be the ability to cope with interruption, prioritize task switching, and return seamlessly to the original task. The ability to switch controlled tasks intelligently with minimal error is central to an emergency physician’s performance. Interruptions are inherent aspects of emergency services, and like police radio dispatchers who have to quickly differentiate and interrupt routine calls to give the highest priority to emergency calls [20], the need for immediate task prioritization and rapidly shifting work demands is the major difference between emergency physicians and office-based primary care physicians. In keeping with this current theory, the definition of multitasking in the EM practice has been updated to include the ability to handle interruptions and task switching.

When EM physicians are interrupted, they shift their attention from the primary task to the interrupting task (e.g., responding to a question from a colleague). If the primary task is an *automatic process,* such as suturing, the physician may well be able to answer the colleague and suture simultaneously [21]. However, if the primary task is a *controlled task*, which utilizes the same cognitive domain as the interrupting task (e.g., taking a history from the patient and answering a colleague’s question), tasks are stacked and the physician has to prioritize whether to switch tasks [22]. If he decides to interrupt the history taking and answer the colleague’s question, he relies on short-term memory and vigilance to return to where he left off in the history taking. If there are multiple stacked tasks, short-term memory and vigilance degrade, making it more difficult for the physician to return to the original task.

In addition, an unsolicited interruption may occur, where as a result of situational awareness, the physician interrupts his own primary task to attend to a more urgent task (e.g., stopping history taking to prevent a confused geriatric patient from falling out of bed). Such interruptions are obviously beneficial, and EM residents need to develop situation awareness when managing the ED.

As mentioned, not all interruptions are harmful, and the consequences of interruptions are myriad, ranging from trivial and innocuous (e.g., temporary distraction) to potentially lifesaving (e.g., responding promptly to a telemetry arrhythmia alarm). At the other extreme, more serious medical errors occur when concentration is broken during crucial tasks [23] (e.g., administering a wrong dose of intravenous chemotherapy). In the aviation industry, interruptions and distractions are one of the most common causes of pilot error, and lapses of attention where a pilot is preoccupied with one task to the exclusion of another have contributed to many aviation accidents [24].

Clearly, coping with components of multitasking such as interruption and break-in-tasks is a major part of an EM resident’s development. This skill needs to be properly taught and evaluated, and residents need to understand the decision-making process when experts juggle between multiple tasks. EM faculty need to know how to remediate any identified deficiencies in multitasking ability.

#### 1.4.1 Methods of teaching multitasking

Role modeling is traditionally the method of choice for learning multitasking skills. As self-directed learners, EM residents opportunistically observe and reflect on positive and negative examples of multitasking and internalize the coping mechanisms faculty employ when dealing with the cognitive load of multiple stacked tasks. The faculty need to be motivated to recognize and capitalize on such teachable moments. Arguably, such real-time teaching will itself involve interruption and multitasking to set aside time to debrief the resident. When done in a timely fashion, along with the contextual nature of such concrete experiences, residents learn indelible lessons.

EM residency programs often lack a formalized curriculum that teaches pitfalls of task switching, task stacking, and the over reliance on short-term memory and vigilance [22]. Residents need to learn that when faced with a cognitive load caused by an interruption, one’s natural tendency is to fall back on heuristics and such decision-making biases can influence the iterative process that leads to assimilation of new important information, which may influence patient care outcomes. For example, a resident taking history from a patient may fall prey to a confirmation or anchoring bias in order to expedite a return to his multiple stacked tasks, while an experienced faculty with insight into such biases may know when to deliberately slow down, pay attention, and delegate non-urgent tasks.

**Figure 1:**
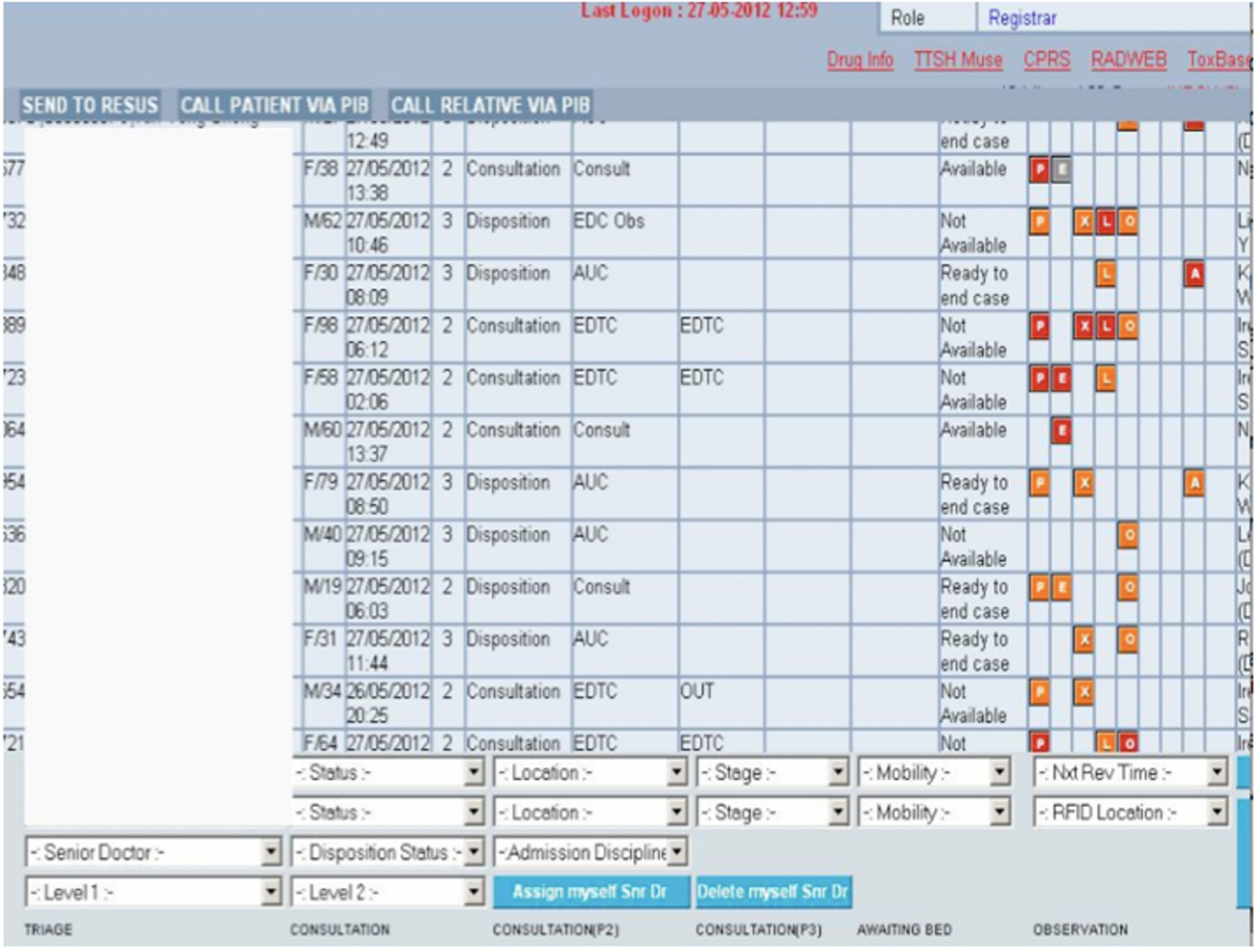
**Screen snapshot of an emergency medicine record system.** Visual cues on an EMR system highlight unreviewed investigation results (P, point of care; X, X-ray; E, EKG; in red), patient location, and patients awaiting disposition.

Residents should be taught interruption-handling strategies like when to ignore the interruption or how to suspend a primary task in a state that makes it easy to return to and how to resume a previously interrupted task [25],[26]. They should be explicitly taught recall techniques (e.g., visual cues, making a short note, involving nursing staff as a backup reminder). Where possible, technological solutions should be employed to aid physicians in recall and decision-making [27]. Figure 1 shows a screenshot of the electronic EM record system, EDWeb®, which provides visual cues on outstanding tasks. The goal is for residents to know when to avoid certain interruptions which would be detrimental to their work processes, while maintaining situational awareness and keeping an open eye or ear to know when switching tasks is appropriate to prioritize another patient over the current one. These skills are critical to the successful management of multiple patients.

## 2 Conclusions

Interruptions are merely indicative of the need for constant communication and coordination in the complex ecosystem that is healthcare [28]. Interruptions and the ability to multitask is an integral part of the EM practice and one of the most important skills to acquire during residency.

There should be a formal curriculum to teach EM residents about the human factors framework and the pitfalls of multitasking and develop insight into personal biases. Residents should be taught techniques to minimize errors when encountering interruptions, and where possible, electronic decision support tools should be employed to aid the clinicians. Techniques in multitasking, team management, delegation of duties, and resource utilization can be learnt by faculty modeling, practiced in a simulation setting using multiple patient encounters and refined in an actual clinical practice. The faculty should be cognizant that residents need to be placed in an environment suitable to their multitasking ability and that frequent formative evaluations will guide their deployment to progressively more complex environments. Evaluation of multitasking ability is best conducted by direct observation using the established milestones.

## Competing interests

The author declares that he has no competing interests.
